# Easy and Fast Discrimination of Female Sand Flies from *Lutzomyia* Species with Infrared Spectroscopy and Multivariate Analysis

**DOI:** 10.1021/acs.analchem.5c00579

**Published:** 2025-05-27

**Authors:** Matheus E. P. Barbosa, Miller Lacerda, Camila Calvani, Thiago Franca, Aline E. Casaril, Jucelei O. M. Infran, Alessandra G. Oliveira, Cicero Cena

**Affiliations:** † Programa de Pós-Graduação em Doenças Infecciosas e Parasitárias, Faculdade de Medicina, 54534 UFMS-Universidade Federal de Mato Grosso do Sul, Campo Grande, MS 79070-900, Brazil; ‡ Laboratório de Parasitologia Humana, Instituto de Biociências, 54534 UFMS-Universidade Federal de Mato Grosso do Sul, Campo Grande, MS 79070-900, Brazil; § Laboratório de Fotodiagnóstico (SISFOTON-UFMS), 54534 UFMS-Universidade Federal de Mato Grosso do Sul, Campo Grande, MS 79070-900, Brazil

## Abstract

Accurate identification of sandfly species is critical for controlling and preventing the spread of visceral leishmaniasis, a major public health concern in Latin America. Morphological similarities between female Lutzomyia cruzi and Lutzomyia longipalpis present a significant challenge for traditional identification methods, highlighting the need for innovative alternative approaches. This study evaluates the potential of Fourier transform infrared (FTIR) spectroscopy associated with principal component analysis (PCA) and machine learning (ML) algorithms for species discrimination. Using vibrational bands predominantly assigned to lipid and carbohydrate molecules, the method achieved over 95% classification accuracy with the Linear support vector machine. Our results demonstrate that the 2970–2800 cm^–1^ (C–H stretching) and 1154–1109 cm^–1^ (C–O and C=C stretching) spectral ranges are particularly informative for distinguishing the species. The approach offers a rapid, cost-effective, and nondestructive solution for entomological classification, significantly enhancing vector surveillance capabilities. The integration of FTIR and machine learning (ML) techniques represents a transformative tool for entomological and epidemiological studies, providing valuable support for disease control strategies.

## Graphical abstract

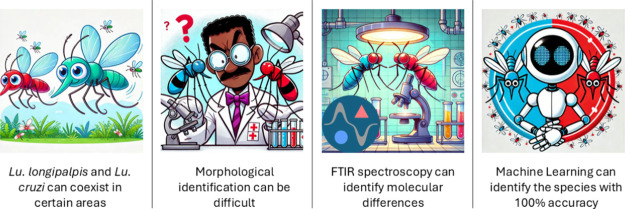

## Introduction

The One Health approach promotes a systemic and integrated view of health, essential for addressing the complex global challenges of the 21st century. It simultaneously fosters sustainable scientific, economic, and social advancements. Within this perspective, the accurate and efficient identification of vector insect species is fundamental for the control and prevention of diseases.^1^

Among the main tropical diseases in Latin America, visceral leishmaniasis—the most severe form of leishmaniasis—stands out due to its high occurrence. In 2021, 93.5% of the reported cases were registered in Brazil, highlighting its prevalence in the region. This disease also represents a significant health challenge for other countries in the Global South.^2^ Leishmaniasis is caused by protozoan pathogens of the genus *Leishmania*, which have a heterogeneous lifecycle involving mammalian hosts and sandfly vectors.^3^ Females of Lutzomyia cruzi and Lutzomyia longipalpis are competent vectors of *Leishmania infantum*, the etiological agent of visceral leishmaniasis in the Americas^4–6^

Lutzomyia longipalpis is widely distributed across countries in the Americas, whereas Lutzomyia cruzi is confined to Bolivia and certain regions of Brazil.^7^ Females of both species exhibit generalist feeding behaviors, feeding on humans, domestic, and wild animals.^8–10^ Lutzomyia longipalpis and Lutzomyia cruzi are sympatric species that coexist in the same geographical areas and exhibit significant morphological similarities,^11–13^ which impose difficulties on their identification by traditional morphological analysis.

Precise identification of sandfly species is crucial for monitoring their geographic distribution and population density, which are essential for understanding the transmission patterns of leishmaniasis and enabling the implementation of targeted control strategies.^7^ Moreover, species identification helps assess vector competence, i.e., the ability of insects to transmit pathogens.^14^

Traditional sandfly identification relies on morphological and taxonomic methods, requiring detailed analysis of anatomical features such as cibarial morphology, spermathecae, and palps.^13^ While dichotomous keys are widely used, they often depend on features that may be lost or obscured during sample preparation, leading to potential misidentifications.^14,15^ The morphological similarity between species adds to this complexity, particularly in females, which often require specialized expertise for accurate classification.^13^

Due to significant genetic polymorphisms among populations,^16^ multiple sibling species, and distinct phenotypes based on abdominal tergite,^17^ populations of Lutzomyia longipalpis are considered a complex of species. Differentiation between female Lutzomyia longipalpis and Lutzomyia cruzi is particularly challenging because they are morphologically indistinguishable, then the development of alternative identification methods becomes necessary.^18–20^

Implementing rapid vector identification methods enhances epidemiological surveillance, allowing for the timely detection of vector-borne disease outbreaks. This capability is crucial in dynamic contexts where transmission patterns may shift due to environmental or social factors.^21^ Simple, user-friendly tools also reduce the reliance on specialized professionals, broadening the capacity for disease surveillance and control. Integrating these methods with artificial intelligence algorithms further facilitates the data collection and analysis of vector presence and distribution. This enables comprehensive risk assessments of zoonotic diseases and contributes to robust early warning systems.

In this context, infrared spectroscopy associated with machine learning algorithms has been successfully applied for species identification, offering a highly accurate method with minimum sample preparation, fast results with low cost, and easy implementation. First, Fourier transform infrared (FTIR) spectroscopy combined with machine learning algorithms was used to identify multidrug-resistant Escherichia coli (E. coli) strains. Its potential was demonstrated by analyzing 80 E. coli samples, 40 standard strains, and 40 multidrug-resistant isolates, with principal component analysis (PCA) and machine learning techniques. An overall accuracy of 75% was achieved for distinguishing between pathogenic and nonpathogenic E. coli strains after proper principal component selection.^22^

The Fourier transform infrared (FTIR) spectroscopy combined with machine learning (ML) successfully differentiates Brachiaria brizantha seed cultivars and assesses seed vigor. The molecular vibrational modes showed key differences in biochemical compounds such as lipids and proteins, which are pivotal for classification. Vibrational bands around 1748 and 1543 cm^–1^ were particularly significant for distinguishing high- and low-vigor seeds. This approach not only enhances the efficiency of seed identification but also provides a rapid and accurate alternative to traditional, labor-intensive methods.^23^

Wood samples can be easily distinguished by considering the different species within the same family (Eucalyptus) as well as those from various families of native Brazilian wood. Besides the high concentration of cellulose, lignin, and hemicellulose in the samples, the FTIR technique associated with PCA was able to clearly distinguish between the species by assessing small differences in the spectra due to minor extractive components in the wood samples.^24,25^

These subtle changes in the infrared spectra assigned to minor molecular components also allowed the identification of Lutzomyia longipalpis and Lutzomyia cruzi males according to their biomes. In the study, Fourier Transform Infrared Photoacoustic Spectroscopy (FTIR-PAS) combined with principal component analysis (PCA) and machine learning achieved 100% classification accuracy in distinguishing *Lutzomyia* populations based on protein content variations. By providing a rapid, cost-effective, and nondestructive alternative, this approach offers a powerful auxiliary tool for species identification, crucial for understanding vectorial capacity and implementing effective disease control strategies.^26^

Given the challenges posed by the morphological complexity in distinguishing female specimens of Lutzomyia longipalpis and Lutzomyia cruzi and recognizing that these species likely exhibit distinct molecular compositions due to inherent genetic differences, this study investigates the application of Fourier transform infrared (FTIR) spectroscopy combined with principal component analysis (PCA). Our approach aims to assess intraspecies similarities while emphasizing interspecies differences among the specimens. Furthermore, we propose leveraging machine learning algorithms to automate the classification process. We evaluate the predictive performance of the developed model, discuss potential challenges, and highlight the method’s broader applicability and limitations.

## Materials and Methods

### Study Area, Period, and Sampling Identification

The sandflies were collected using CDC (Center for Disease Control) light traps in partnership with the State Coordination for Vector Control. The species were collected from January to December 2022 in the municipalities of Aquidauana and Corumbá, in locations previously reported for the prevalence of Lutzomyia cruzi in Corumbá and Lutzomyia longipalpis in Aquidauana. Corumbá (19°00′52″ S, 57°39′20″ W) and Aquidauana (20°28′08″ S, 55°47′58″ W) are municipalities in the state of Mato Grosso do Sul, Brazil. Corumbá lies along the Paraguay River, within the Pantanal region, a globally significant wetland ecosystem. Aquidauana, located approximately 140 km east of Corumbá, is a gateway to the southern Pantanal, which is influenced by its biodiversity and ecological significance. Identification of the specimens was based on their place of origin and the morphology of their spermatheca, following the identification procedure by Galati (2018)^13^ as observed from the cut of the last two tergites of the abdomen.

Females without eggs and not engorged with blood were selected for identification by cutting the last two tergites of the abdomen. Phlebotomine species were identified based on the morphology of their spermatheca and place of origin. After visualizing the spermatheca, the female bodies were stored in an Eppendorf tube with 70% alcohol. The phlebotomine bodies were dried at 60 °C for 5 min in a drying oven to maintain sample integrity and avoid damage.

### Data Collection-FTIR Measurements

The sandfly specimens are tiny, around 2 mm long. Here, to improve the signal-to-noise ratio, 120 specimens of each species were measured using four entire sandfly specimens in the sample holder. The four whole Lutzomyia sandfly specimens were placed directly onto the ATR sample holder without pulverization or mixing. A Germanium ATR crystal was used to ensure direct contact with the infrared beam. Using four specimens improved the signal-to-noise ratio, as individual sandflies (∼2 mm) produced weak signals. This approach enhanced the spectral intensity, minimized individual variability, and ensured reproducibility. The ATR sample holder consists of a standard plate with an optical window and a pressure device on top (Figure 1), which ensures optimal contact between the sample and the crystal, enhancing signal acquisition and improving spectral quality. Then, 30 average spectra of data for each species were obtained by using an attenuated total reflectance accessory (ATR) at a Fourier Transform Infrared Spectrophotometer (Spectrum 100, PerkinElmer). The spectra were collected from 4000 to 600 cm^–1^, with 4 cm^–1^ resolution and 10 scans.

**Figure 1:**
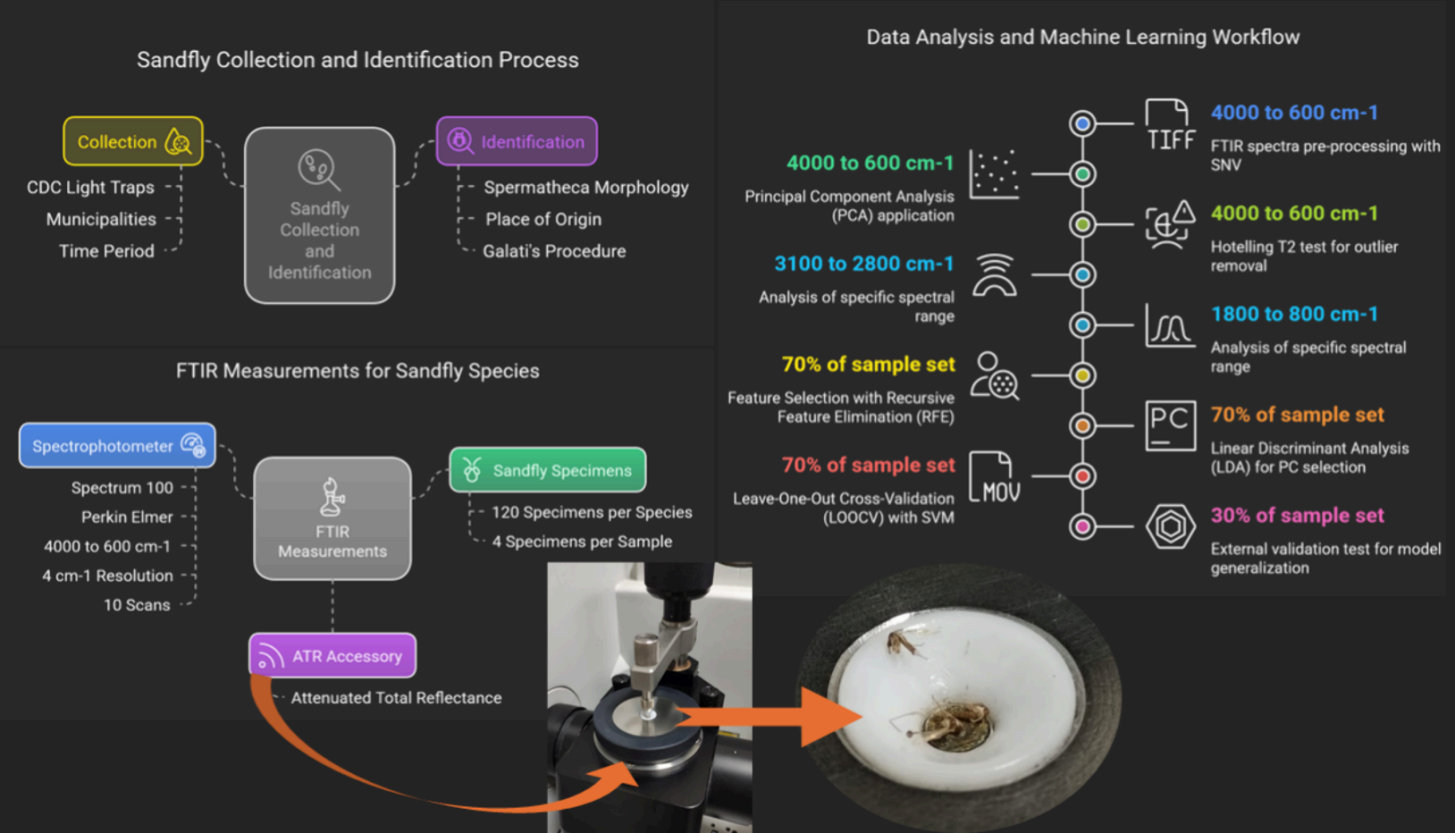
Experimental Workflow for classifying Lutzomyia sandflies using FTIR spectroscopy and machine learning. Female specimens were collected in Corumbá and Aquidauana (MS, Brazil) with CDC light traps and identified based on the spermatheca morphology. Four whole sandflies were placed directly on a germanium ATR crystal for spectral acquisition (detailed photo inset). Spectra were preprocessed with SNV and analyzed using PCA. Principal components were selected via RFE based on LDA performance. Classification models were built with SVM and validated using LOOCV and an external test set (30%).

### Data Analysis and Machine Learning Algorithms

The data analysis was performed in Python (version 3.9.12) using the Scikit-learn package (version 1.1.2).^27^ First, the average FTIR spectra were subjected to the Standard Normal Variate (SNV) preprocessing method, which removes the variation from the baseline and rescales the spectral intensity to prevent interference in the data analysis due to random experimental variations.^28^

The average FTIR-SNV spectra for L. cruzi and L. longipalpis group, in the 4000–600 cm^–1^ range, were submitted to principal component analysis (PCA).^29^ PCA is an unsupervised method that will project our preprocessed data set into a new dimension (PCs – principal components), which aims to maximize the data variance; this dimensionally reduced data set retains most of the information from the original variables and shows how each data sample is distributed in this new dimension (score plot), allowing clustering the similar samples and distinguish groups. Each PC represents a percentage of the data variance and will enable us to analyze the main spectral range that contributes most to the data variance percentage through the loading plot. PCA is an essential step in visualizing the group classification tendency. Then, the Hotelling T2 test was performed to remove outliers; no outliers were found to be removed.^30^ Here we analyzed three different ranges: (i) 4000–600 cm^–1^; (ii) 3100–2800 cm^–1^, and (iii) 1800–800 cm^–1^. This strategy aims to use only those vibrational modes that improve the group clustering and classification and eliminate highly correlated data.

The sample classification was performed by prediction models built by machine learning (ML) algorithms using PC output data from 70% of the sample set. Before sample classification tests, we determined the ideal number of PCs to avoid overfitting and underfitting.^31,32^ Here, we used the Feature Selection Recursive Feature Elimination (RFE), which selects the main PCs that contribute to achieving high accuracy and removes other PCs with the weakest contribution to correct sample classification in ML tests.^33^ The use or removal of a determined PC was made based on the accuracy achieved using Linear Discriminant Analysis (LDA) to classify the samples in a leave-one-out cross-validation (LOOCV) test.

In a brief description, Discriminant Analysis (DA) classifies the sample based on the distance between the sample data and the contour built by using a linear (L) or quadratic (Q) function to separate the classes (groups).^34^ In LOOCV, one sample is taken from the data set, and the others are used to build the prediction model (training). Then, the prediction model accuracy is tested by using the sample data withdrawn from the dataset. The procedure is repeated until all sample data have been tested.^35^

After determining the ideal number of PCs and which PCs most contribute to sample classification in each spectral range analyzed, a LOOCV test was performed by using the respective RFE-PCs data for each range based on a support vector machine (SVM), which organizes each sample class through the optimization of a hyperplane; the hyperplane can be linear or nonlinear, being optimized to reach high performance–between the classes.^36^ Finally, we determined the best spectral range, SVM function (linear, quadratic, and cubic), and PCs to build a predicting model, whose ability for generalization was tested in an external validation test using 30% of the sample set.

## Results and Discussion

Figure 2 shows the sand fly FTIR-SNV spectra for L. cruzi and L. longipalpis species. The line represents the group average FTIR-SNV spectra; the respective standard deviations are shown as a shadow around it. Besides the high similarity between both spectra, they exhibit the same vibrational band position and intensity. A careful examination of the spectra reveals subtle changes around the wider 3270 cm^–1^ band, which is more intense and exhibits a shoulder at 3391 cm^–1^, followed by more intense 2926, 1154, and 1109 cm^–1^ centered bands for L. longipalpis species. These small details can contribute to species differentiation with proper data analysis.

**Figure 2:**
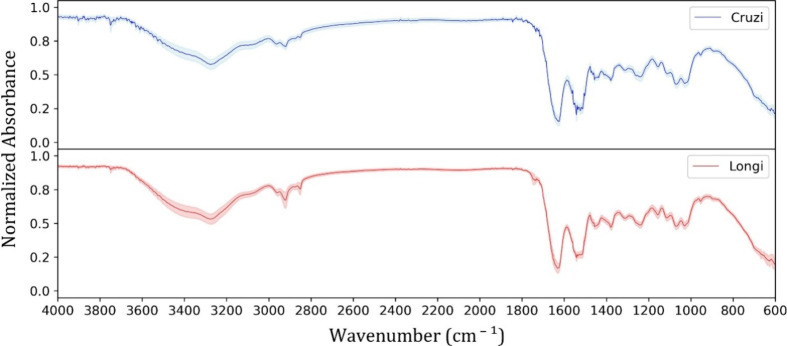
Sandflies averaged FTIR-SNV (solid line) and standard deviation (shadow). Lutzomyia cruzi (blue trace “Cruzi”) and Lutzomyia longipalpis (red trace “Longi”).

The vibrational wide band centered around 3270 cm^–1^ is usually assigned to H-bonded modes from chitin, polysaccharides, proteins, and residual water.^26,37^ The next four medium vibrational bands in the 2970–2800 cm^–1^ range are assigned to C–H stretching from lipids and fatty acid molecules.^26,37^ The high-intensity 1630 cm^–1^ band assigned to C=C and C=O stretching is related to Amide I from proteins, and the 1500 cm^–1^ band assigned to N–O stretching and N–H bending is related to Amine groups.^37,38^ Finally, three medium band groups: (i) 1444 and 1372 cm^–1^ assigned to C–H bending from lipids, proteins, and fatty acids; (ii) 1308 and 1236 cm^–1^ assigned to P=O, O–H bending, C–O stretching, and C–N stretching (Amine) from phospholipids, lipids, and proteins; (iii) 1154, 1109, 1064, 1027 cm^–1^ assigned to C–O stretching, CO–O–CO stretching, and C=C bending from carbohydrates.^37,38^

The FTIR-SNV principal component analysis results are summarized in Figure 3. The score plot for L. cruzi and L. longipalpis could clearly distinguish the groups with a relatively large frontier of separation using only PC1 and PC2 at the 4000–600 cm^–1^ range, which is responsible for 68% of data variance, Figure 3a. The loadings describe the correlation between the principal components and the original FTIR-SNV data. For PC1 at 4000–600 cm^–1^ range, we can observe a major data variance related to the wide and intense 3270 cm^–1^ centered band, followed by a small contribution of three vibrational bands in the 2970–2800 cm^–1^ range from lipids and fatty acids molecules, and the 1154 and 1109 cm^–1^ centered bands as expected from the visual inspection of the FTIR-SNV spectra. Additionally, PC2 also shows the contributions of the amide and amine bands at around 1626 and 1500 cm^–1^, respectively.

**Figure 3:**
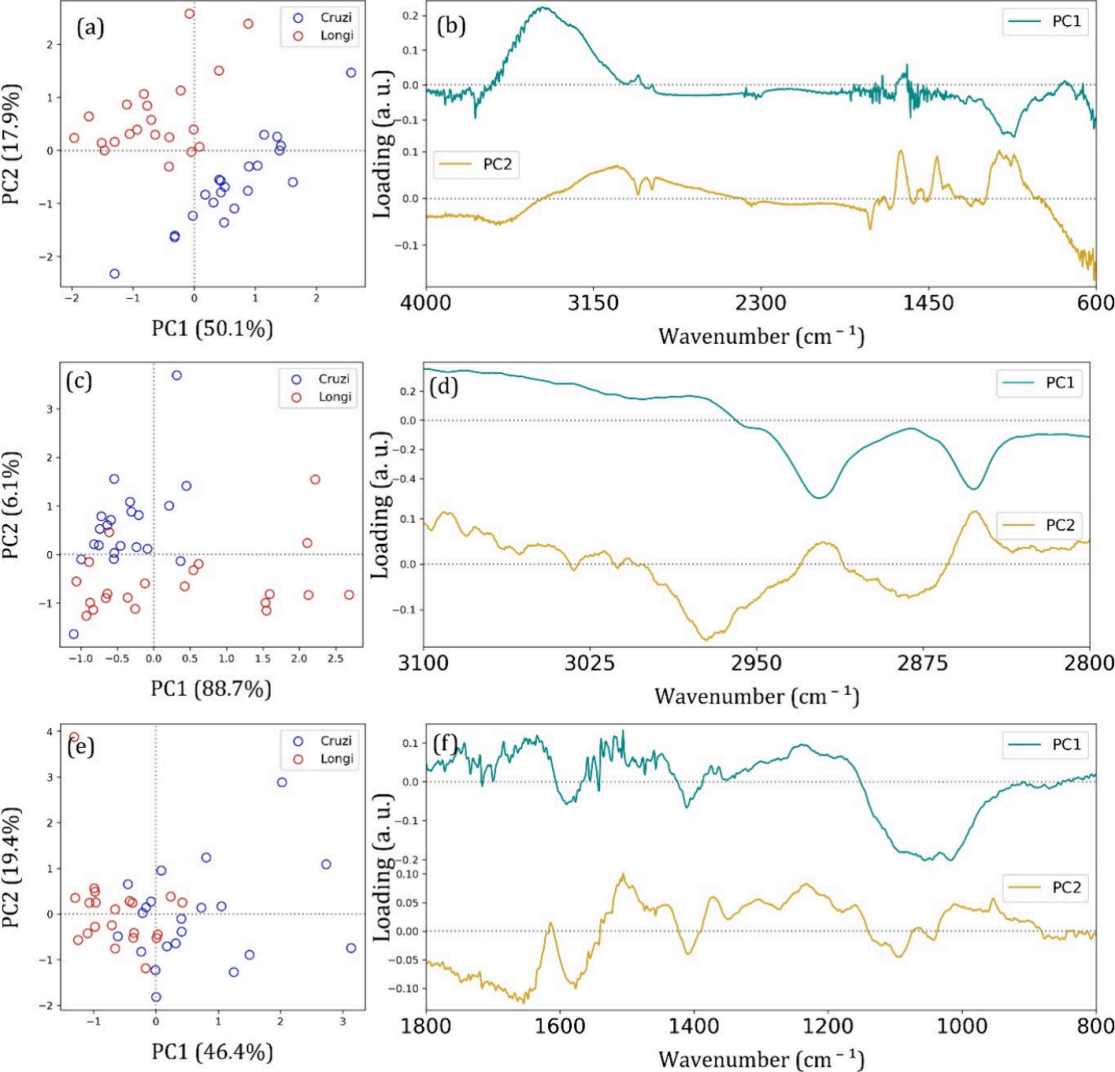
Score plot (a,c,e) and loadings (b,d,f) from PCA data analysis. (a,b) entire spectral range from 4000 to 600 cm^–1^, (c,d) from 3000 to 2800 cm^–1^, and (e,f) from 2000 to 800 cm^–1^. Lutzomyia cruzi (blue circles) and Lutzomyia Longipalpis (red circles).

The wide, strong band centered around 3270 cm^–1^ results from many superimposed contributions, as mentioned before, among them the O–H vibrational mode from water molecules, which cannot be the main data for group selection. Besides the previous sample dehydration, some water molecules can be trapped inside the specimen. Then, we explored other spectral ranges to observe the other FTIR-SNV vibrational bands’ contributions to sample classification. The first analysis was conducted in the 3100–2800 cm^–1^ range, Figure 3b, mainly assigned to C–H stretching from lipids and fatty acids. The score plot for PC1xPC2 is responsible for 94.8% of the data variance, and a reasonable group separation can be achieved. Despite some overlap, the clustering pattern suggests distinct molecular signatures between the two species. The presence of well-defined clusters in the score plot supports the hypothesis that their biochemical compositions differ, particularly in lipid and carbohydrate vibrational modes. The loading shows the main contributions of 2850 and 2923 cm^–1^ bands for PC1 and 2965 cm^–1^ for PC2.

A similar difficulty in clustering both groups using only PC1 × PC2 was found in the 1800–800 cm^–1^ range, in which the main vibrational modes are related to proteins, carbohydrates, and lipid molecules. The score plot PC1 × PC2 is responsible for 65.8% of data variance; a separation tendency between both groups can be observed, but the number of samples embedded in the other group increased. The loading plot shows that the main contributions for data variance are centered around 1600, 1400, and 1100 cm^–1^, assigned to Amide I, C–H bending and C–O stretching from proteins, lipids, and carbohydrates, respectively.

Besides, PC1xPC2 projection could not distinguish between both groups successfully; other principal component projections can improve the results, and we may explore this possibility when using machine learning algorithms. Here, we use the RFE algorithm to select the main principal components with the highest contribution for group selection. The 4000–600 cm^–1^ range used only 3 PCs (1, 2, and 4), the 3100–2800 cm^–1^ range used 6 PCs (1, 2, 4, 5, 6, and 7), and the 1800–800 cm^–1^ range used 10 PCs (1, 3, 4, 5, 6, 7, 8, 10, 11, and 19). The PCA data corresponding to each RFE-PC was submitted to the support vector machine (SVM) algorithm to build a prediction model based on the Linear, Quadratic, and Cubic functions. Figure 4 summarizes the overall accuracy achieved by each prediction model in the LOOCV test for each spectral range analyzed.

**Figure 4:**
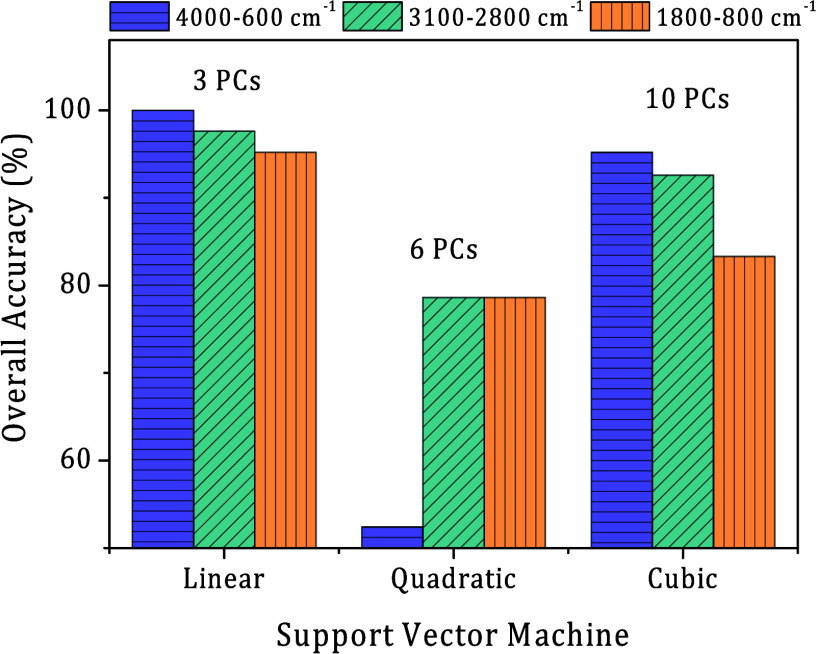
Prediction model overall accuracy for Linear, Quadratic, and Cubic SVM achieved in the Lutzomyia cruzi and Lutzomyia longipalpis samples classification. Different spectral ranges were tested: entire spectral range from 4000 to 600 cm^–1^ (navy bars with horizontal line pattern) using 3 PCs (1, 2, and 4); 3000–2800 cm^–1^ (green bars with right inclined line pattern) using 6 PCs (1, 2, 4, 5, 6, and 7); and 1800–800 cm^–1^ (orange bars with vertical line pattern) using 10 PCs (1, 3, 4, 5, 6, 7, 8, 10, 11, and 19).

The best overall accuracy, exceeding 95%, was achieved by a linear support vector machine (L-SVM) across all spectral ranges. A marginal reduction in overall accuracy, from 100% to 97.6%, was observed as the spectral range transitioned from 4000 to 600 cm^–1^ to 3100–2800 cm^–1^, then to 95.2% in the 1800–800 cm^–1^ range. This small decrease in the results suggests it is statistically irrelevant due to the number of samples tested, because a 5% decrease represents only 1 sample misclassified in our sample set. Then, it also suggests that any spectral range can be used to obtain a good prediction model for laboratory routine use since the correct PCs are selected for this task. However, to achieve the best overall accuracy, using quadratic and cubic SVM was necessary to apply 6 and 10 PCs, respectively. The maximum achieved was 95.2% for Cubic SVM with 10 PCs in the 4000–600 cm^–1^ range.

A confusion matrix (merit figure) illustrates the method’s reliability, which describes how our prediction methods classify the samples according to their real labels; Figure 4. The prediction model is built based on the sample classification in the internal test (LOOCV), based on the algorithm performance with a proper choice of PCs. The left side of Figure 5 describes the same accuracy values shown in Figure 4 for Linear SVM in the different spectral ranges at the LOOCV test. The right side in Figure 5 shows the accuracy values achieved in the validation test for the Linear SVM using the proper PCs for each spectral range determined in the LOOCV test.

**Figure 5:**
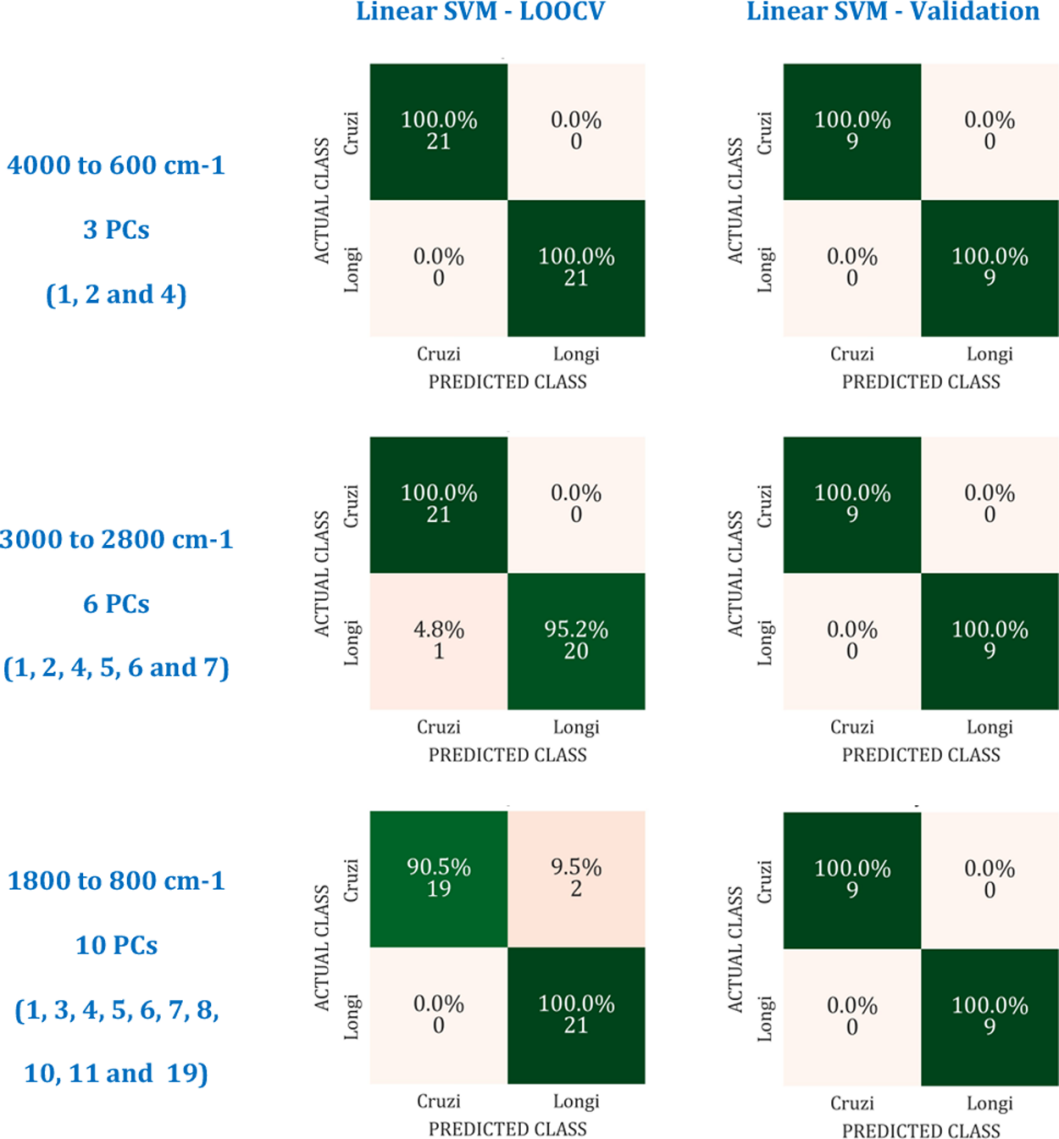
Confusion Matrices for LOOCV test (left side) and Validation test (right side) using the Linear SVM algorithm. Sample (Lutzomyia cruzi and Lutzomyia longipalpis groups) classification demonstrated for the entire spectral range from 4000 to 600 cm^–1^ using 3 PCs (1, 2, and 4); from 3000 to 2800 cm^–1^ using 6 PCs (1, 2, 4, 5, 6, and 7); and from 1800 to 800 cm^–1^ (1, 3, 4, 5, 6, 7, 8, 10, 11, and 19).

The validation tests were successful, with 100% overall accuracy for all spectral ranges analyzed with the proper choice of PCs. A small deviation between the overall accuracy achieved in the LOOCV and validation test is observed, but it is only due to the difference in the number of samples used for each test, 70% in LOOCV and 30% in validation. The results suggest that, besides the great contribution of the 3270 cm^–1^ centered band for group clustering, it is not decisive for the result. We can also apply the same algorithm (Linear SVM) and number of PCs (1, 2, and 4) in other spectral ranges and achieve a great prediction model with an overall accuracy above 95%.

Numerous challenges arise in phlebotomine taxonomy regarding accurate taxonomic identification. These challenges encompass technical variables such as capture, transportation, clarification, and mounting techniques, in addition to those associated with the insect’s morphological characteristics. A subset of these morphological traits remains undetectable with the available diagnostic tools, potentially resulting in misidentifications.^13,15^ This methodology overcomes the challenges posed by Lutzomyia longipalpis and Lutzomyia cruzi females’ morphological similarities, providing a rapid and cost-effective alternative for species-level identification.

## Conclusions

This study demonstrated the effectiveness of Fourier-transform infrared (FTIR) spectroscopy combined with principal component analysis (PCA) and machine learning (ML) algorithms for the rapid and accurate discrimination of female Lutzomyia cruzi and Lutzomyia longipalpis. Despite their high morphological similarity, distinct biochemical signatures were identified, particularly in spectral regions associated with lipids and carbohydrates (2970–2800 and 1154–1109 cm^–1^). The classification model, based on a linear support vector machine (SVM), achieved an accuracy exceeding 95%, confirming the robustness of the proposed approach.

A key advantage of this method lies in its speed, cost-effectiveness, and nondestructive nature, making it a promising alternative to traditional morphological identification, which requires specialized expertise and is susceptible to misclassification. The selection of principal components (PCs) was optimized using recursive feature elimination (RFE), ensuring that only the most informative features contributed to the model, thereby reducing the risk of overfitting.

While PCA component selection is commonly guided by the cumulative variance explained—often using the “elbow” criterion—it is important to emphasize that this criterion alone does not always yield the most effective classification outcome. Early PCs may capture a high variance driven by noise or background variation unrelated to class separation. Consequently, selecting components solely based on variance may overlook subtle, class-discriminative features embedded in higher-order PCs.

To address this, we adopted a hybrid strategy. For the full spectral range (4000–600 cm^–1^), the first three PCs—accounting for the greatest variance—were sufficient to achieve high classification accuracy. However, when the spectral window was narrowed, leading to a reduction in vibrational mode information, it became necessary to incorporate higher-order PCs that explained less variance individually. Despite their lower variance contribution, these PCs captured nuanced spectral differences that enhanced the class separation.

Internal validation using leave-one-out cross-validation (LOOCV) confirmed the stability of the models with no indication of overfitting. The consistent performance across validation sets suggests that even PCs with a low variance can carry biologically meaningful and class-relevant information. Therefore, a classification-driven approach—balancing predictive accuracy with variance monitoring—is both robust and justifiable, particularly when working with complex spectral data sets.

Unlike conventional PCA strategies that rely solely on explained variance, our approach prioritized components that improved the classification performance. This underscores the value of combining variance analysis with predictive accuracy optimization.

Furthermore, the study evaluated different spectral ranges and their impact on the classification performance. While the full range (4000–600 cm^–1^) yielded the highest accuracy, comparable results were obtained with narrower spectral intervals. This flexibility indicates that the methodology can be adapted to varying experimental conditions and instrumental limitations.

Future work should expand the data set to include additional vector species, test alternative ML algorithms, and validate the model across broader geographic regions and environmental conditions. Moreover, exploring the biochemical basis of the observed spectral differences may provide deeper insight into the mechanisms underlying species differentiation.

By integrating vibrational spectroscopy with machine learning, this study offers a powerful tool for enhancing vector identification and epidemiological monitoring with broad applicability in entomology and infectious disease control.
